# Optic Disc Drusen in Pseudoxanthoma Elasticum Are Associated with the Extent of Bruch’s Membrane Calcification

**DOI:** 10.3390/jcm13123395

**Published:** 2024-06-10

**Authors:** Kristin Raming, Sandrine H. Künzel, Maximilian Pfau, Doris Hendig, Frank G. Holz, Kristina Pfau

**Affiliations:** 1 Department of Ophthalmology, University of Bonn, 53127 Bonn, Germany; 2 Department of Ophthalmology, University of Basel, 4031 Basel, Switzerland; 3 Institute of Molecular and Clinical Ophthalmology Basel, 4031 Basel, Switzerland; 4 Institute for Laboratory and Transfusion Medicine, Heart and Diabetes, Center North Rhine-Westphalia, University Hospital of the Ruhr University of Bochum, 32545 Bad Oeynhausen, Germany

**Keywords:** pseudoxanthoma elasticum, PXE, optic disc drusen, angioid streaks, Bruch’s membrane

## Abstract

**Background/Objectives**: To assess the frequency, extent, localization and potential progression of optic disc drusen (ODD) and the correlation with the angioid streak (AS) length and retinal atrophy in patients with pseudoxanthoma elasticum (PXE). **Methods**: This retrospective study included patient data from a dedicated PXE clinic at the Department of Ophthalmology, University of Bonn, Germany (observation period from February 2008 to July 2023). Two readers evaluated the presence, localization, and the extent of the ODD on fundus autofluorescence (FAF) imaging at baseline and the follow-up assessments. Additionally, we measured the length of the longest AS visible at baseline and follow-up and the area of atrophy at baseline, both on FAF. **Results**: A total of 150 eyes of 75 PXE patients (median age at baseline 51.8 years, IRQ 46.3; 57.5 years, 49 female) underwent retrospective analysis. At baseline, 23 of 75 patients exhibited ODD in a minimum of one eye, resulting in an ODD prevalence of 30.7% in our cohort of PXE patients. Among these, 14 patients showed monocular and 9 binocular ODD that were localized predominantly nasally (46.9%). During the observational period (mean 97.5 ± 44.7 months), only one patient developed de novo ODD in one eye and one other patient showed a progression in the size of the existing ODD. The group of patients with ODD had significantly longer ASs (median 7020 µm, IQR 4604; 9183, vs. AS length without ODD: median 4404 µm, IQR 3512; 5965, *p* < 0.001). No association with the size of the atrophy was found at baseline (*p* = 0.27). **Conclusions**: This study demonstrates a prevalence of ODD of 30.7%. ODD presence is associated with longer ASs (an indicator of the severity and extent of ocular Bruch’s membrane calcification), suggesting that ODD formation is tightly related to ectopic calcification—possibly secondary to calcification of the lamina cribrosa. Prospective studies investigating the impact of ODD (in conjunction with intraocular pressure) on visual function in PXE warrant consideration.

## 1. Introduction

Pseudoxanthoma elasticum (PXE, OMIM #264800) is an inherited multisystemic disease caused by biallelic mutations in the ABCC6 gene, leading to ectopic calcification of elastic fibers in the skin, the cardiovascular system and the eyes [1,2].

Calcification in PXE is driven by low levels of inorganic pyrophosphate, a key inhibitor of ectopic calcification [3]. The calcification of Bruch’s membrane (BrM) results in a reduction of its elasticity and permeability, leading to a variety of ocular lesions [4,5]. Clinically, the calcification starts around the optic disc and progresses toward the periphery throughout the patient’s life [6]. The transition zone between the (central) calcified BrM and (peripheral) non-calcified area is called the Peau d’orange. In the central zone, angioid streaks (ASs) signify breaks in the calcified, brittle BrM and are considered a phenotypic ocular hallmark of PXE.

Optic disc drusen (ODD) are an additional clinical aspect observed in PXE. The prevalence of ODD is increased in various ocular pathologies, but notably in PXE, with an incidence previously estimated to be 24.5% in PXE patients in a single French cohort [7]. ODD are calcified deposits at the optic nerve head resulting from impaired axonal metabolism and are estimated to have a prevalence of 0.3–2.4% in the general population [8,9]. Mostly manifesting as yellow to white clusters around the optic disc on funduscopy, buried ODD can appear as an elevated disc or blurred disc margin mimicking papilledema. Differentiation is possible through imaging modalities such as enhanced depth imaging optical coherence tomography (OCT), fundus autofluorescence (FAF), or ultrasonography.

Most PXE patients with ODD remain asymptomatic and the eventual visual loss is predominantly caused by exudation secondary to choroidal neovascularization. However, a recent study has shown that thinning of the inner retinal layers is associated with reduced retinal sensitivity in PXE, even in eyes without outer retinal alterations, and case reports of markedly constricted visual fields in PXE patients with ODD were reported [10].

This study aims to provide a comprehensive characterization of ODD in patients with PXE and the association with calcification, leveraging one of the largest cohorts and longest follow-up periods.

## 2. Materials and Methods

### 2.1. Study Design and Population

In this single-center retrospective study, the data of PXE patients who were examined at our department between 19 January 2022 and 19 July 2023 were included in the study. The retrospective analysis extended back to their first visit including 55° FAF imaging. The inclusion criteria were a confirmed diagnosis of PXE according to the criteria of Plomp et al. [11] and a minimum of one follow-up visit. The study adhered to the tenets of the Declaration of Helsinki and was approved by the Ethics Committee of the University of Bonn (lfd. Nr. 316/11). Informed consent was obtained from all the patients.

Each patient underwent an ophthalmic examination, including best-corrected visual acuity testing, dilated funduscopy, spectral-domain OCT with volume scans of 30° × 25° and 55° FAF (both Spectralis, Heidelberg Engineering, Heidelberg, Germany). Other examinations, e.g., color fundus photography (Zeiss Visucam, Carl Zeiss Meditec, Jena, Germany) or visual field testing (Zeiss Humphrey HFA 3/860 Field Analyzer, Carl Zeiss Meditec, Jena, Germany), were performed based on the clinical findings and specific diagnostic questions. Baseline was defined as the first visit date with available bilateral 55° FAF imaging, while the follow-up data included the latest available bilateral 55° FAF.

### 2.2. Imaging Analysis

**Figure 1:**
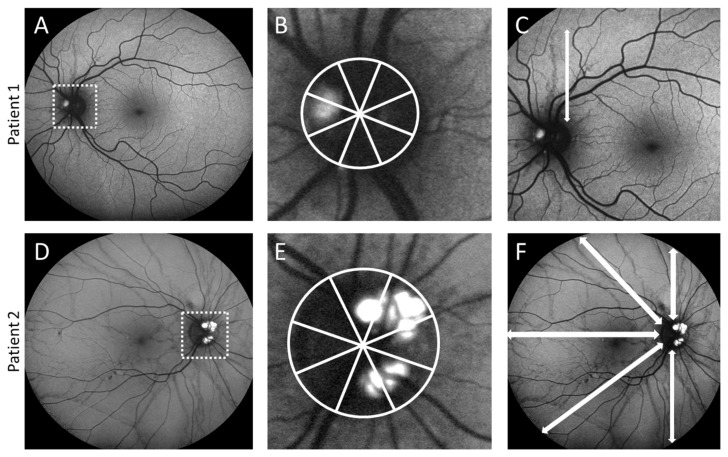
Image analysis: fundus autofluorescence (FAF) images of a 25-year-old (**A**–**C**) and a 28-year-old (**D**–**F**) PXE patient (both female). (**A**,**D**): Dashed line boxes: Snippet of the images shown enlarged in (**B**) and (**E**), respectively. (**B**,**E**): The white overlay shows the grading of ODD regarding their location. (**C**): White arrow indicates angioid streak length, (**F**): Directions of reference lengths if the angioid streak extended the FAF image.

The ODD characteristics were assessed on 55° FAF images (see Figure 1A,B,D,E). If ODD were present, their extent was graded regarding the number of quadrants (1/4, 2/4, 3/4, circular) involved. Additionally, the localization of the ODD was determined by dividing the optic disc into 8 segments (superior, superior-nasal, nasal, inferior-nasal, inferior, inferior-temporal, temporal). If ODD were present in more than one segment, the overall predominantly affected segment was considered the (primary) localization (see Figure 1B,E).

The AS length was assessed on the FAF images using the implemented measurement tool in the Heidelberg Eye Explorer (Heidelberg Engineering, Heidelberg, Germany, Software version 2.6.4) as the distance from the optic disc rim to the end of the AS (see Figure 1C). If the AS extended beyond the 55° FAF image rim nasally, the longest visible AS on the temporal FAF images was measured. Localization of the AS beyond the superior, temporal or inferior regions was recorded with a fixed reference value based on the direction (see Figure 1F). If the AS could not be measured due to progressed atrophy, it was marked as NA.

All the imaging data underwent independent review by two readers (KR and SK). In case of disagreement regarding the presence of ODD, the case was discussed with a senior reader (KP) until a consensus was reached. For the measurements of the AS length, the mean value from both readers was used.

Furthermore, one reader (KR) measured the area of retinal atrophy on the FAF images at baseline using the Region Finder tool (Region Finder Software, Heidelberg Engineering, Heidelberg, Germany Software version 2.6.6.0).

The presence, extent and localization of the optic disc drusen (ODD) were analyzed on the FAF images (Figure 1B: ODD with an extent of 1 quadrant and predominant nasal localization, Figure 1E: ODD with an extent of 3 quadrants and overall nasal localization).

The longest angioid streak (AS) was assessed on the FAF image (see Figure 1C) with measurements of 3852 µm (Reader1) and 3212 µm (Reader 2). If the AS extended beyond the 55° FAF image (see Figure 1F), reference lengths were set for all the directions (superior 5000 µm, superior-temporal 11,000 µm, temporal 11,600 µm, temporal-inferior 11,000 µm, inferior 5700 µm, inferior-nasal 3800 µm, nasal 1800 µm and superior-nasal 3400 µm).

### 2.3. Statistical Analysis

All the statistical analyses were performed using RStudio Version 2022.12.0+353 (R Foundation for Statistical Computing, Vienna, Austria). The categorical variables were described through the absolute (*n*) and relative frequencies (%). In case of non-normally distributed data, the median and the 25% and 75% interquartile ranges (IQR) were assessed. Normally distributed data were described by the mean ± standard deviation.

## 3. Results

### 3.1. Cohort Characteristics

A total of 150 eyes from 75 PXE patients (median age at baseline 51.8 years, IQR 46.3; 57.5, range 14.3–70.1, n = 49, 65.3% female) were included in the study. Retrospective analyses were performed on imaging data from between February 2008 and July 2023, with a mean follow-up time of 97.5 ± 44.7 months. At baseline, at total of 42 of the 75 patients (56%) required previous anti-VEGF treatment due to secondary choroidal neovascularization (CNV).

### 3.2. Optic Disc Drusen Prevalence, and Characteristics

**Figure 2:**
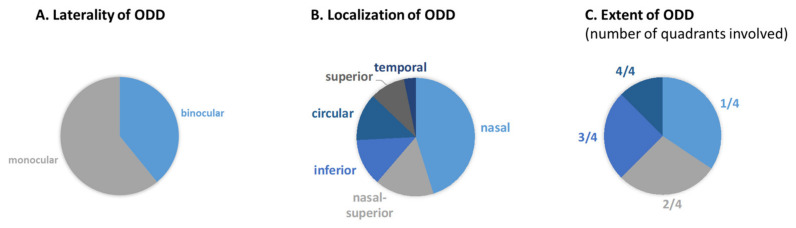
Characteristics of optic disc drusen (ODD) in eyes of patients with pseudoxanthoma elasticum (PXE). (**A**) ODD are present monocularly in 60.9% and binocularly in 39.1% in patients with ODD. (**B**) The majority of ODD are localized nasally. (**C**) Illustration of the extent of the ODD by the number of quadrants involved, which are widely distributed.

Among all 75 patients analyzed at baseline, a total of 23 patients showed ODD on FAF in at least 1 eye, resulting in a prevalence of 30.7%. In all 150 eyes, 32 showed ODD (21.3%), with 19 in the right eyes and 13 in the left eyes. Fourteen patients had unilateral (60.9%) and nine patients (39.1%) bilateral ODD. The ODD were localized nasally in 15 eyes (46.9%), nasal-superior in 5 eyes (15.6%), inferior in 4 eyes (12.5%), superior in 3 eyes (9.4%), temporal in 1 eye (3.1%) and 4 eyes showed circular ODD (12.5%) (see Table 1 and Figure 2).

**Table 1:** Demographic data and characteristics of the optic disc drusen (ODD) and angioid streaks (ASs) in patients with pseudoxanthoma elasticum (PXE) at baseline and follow-up examination.

			Baseline	Follow-Up
Number of PXE patients [*n*]			75	75
Number of eyes [*n*]			150	150
Age [years, median, IQR]			51.8 [46.3; 57.5]	60.7 [54.9; 65.8]
ODD presence [*n*, %]	OD		19 (25.3)	20 (26.7)
	OS		13 (17.3)	13 (17.3)
	total		32	33
	unilateral		14	15
	bilateral		9	9
ODD extent ^1^	OD	1/4	7	7
		2/4	7	8
		3/4	2	2
		4/4	3	3
	OS	1/4	4	4
		2/4	2	2
		3/4	6	6
		4/4	1	1
AS length [µm, median, IQR]	OD		4728.8	5144.5
			[3591.9; 6413.8]	[4151.5; 7821]
	OS		4543.5	5000
			[3586; 7567]	[3434; 7605.9]
	ODD + ^2^		5924	8677
			[4850; 9134]	[5383; 10,925]
	ODD − ^3^		4390	4810
			[3547; 5642]	[3950; 6375]

^1^ number of quadrants involved. ^2^ ODD + considered as the presence of ODD. ^3^ ODD − considered as the absence of ODD

**Figure 3:**
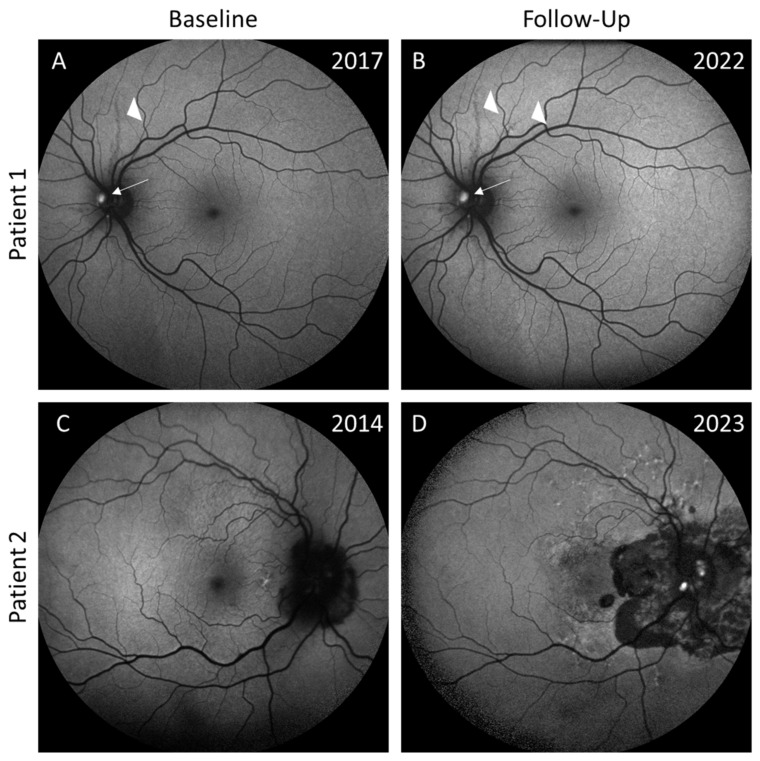
Clinical examples of the progression of optic disc drusen (ODD). (**A**,**B**) show fundus autofluorescence (FAF) images in 2017 and 2022 of a 25-year-old female PXE patient with unilateral ODD, which increased in size (arrow). The longest AS visible stayed stable, but a new AS developed (marked by the two arrowheads). (**C**) illustrates the FAF of a 56-year-old female PXE patient with suspected ODD nasal superior in the right eye. These become visible on FAF t the follow-up visit 7.8 years later (see (**D**)), with additional ODD temporal-inferior. Furthermore, the atrophy progressed markedly in size.

At the final visit, only one female patient showed the development of de novo ODD (see Figure 3C,D). In one other female patient, the ODD had progressed in size (see Figure 3A,B).

### 3.3. Angioid Streak Length Association with Optic Disc Drusen

The AS length was measured in 126 of 150 eyes. In 9 eyes, the ASs were not measurable on the 55° FAF imaging due to a very mild phenotype. In 15 eyes, measurement of the AS length was not possible due to the progressed stages with extensive atrophy.

**Figure 4:**
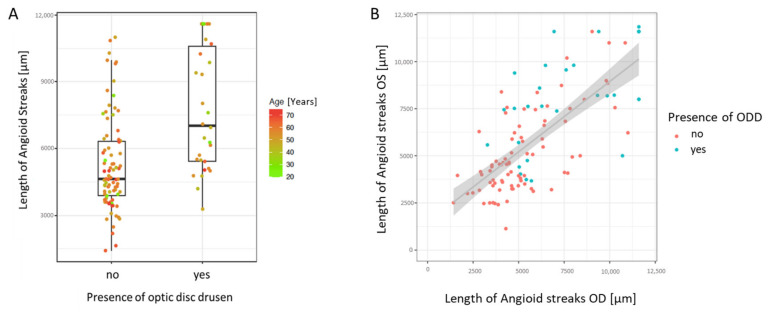
Association of optic disc drusen (ODD) with the angioid streak (AS) length. The ASs are significantly longer in the eyes with ODD (median 7020 µm, IQR 4604; 9183) than the eyes without ODD (median 4404 µm, IQR 3512; 5965, *p* < 0.001), see (**A**). No association with age was found (*p* = 0.37). (**B**) Correlation between the AS lengths of the left and right eyes (*p* = 0.03). Notably, ODD are only visible beyond a distinct AS length of approximately 5000 µm in one eye.

The AS length in the right and left eyes did not differ significantly, with a median length at baseline of 4728.8 µm [IQR 3591.9; 6413.8] in the right eyes and 4543.5 µm [IQR 3586; 7567] in the left eyes, (*p* = 0.19 see Figure 4B).

However, the eyes with ODD had significantly longer ASs (median 5924 µm, IQR 4850; 9134) than the eyes without ODD (median 4390 µm, IQR 3547; 5642, *p* < 0.001) (see Table 1 and Figure 4A). No association with age was found (*p* = 0.37). In general, ODD were mostly present in eyes with an AS length of ≥5000 µm (see Figure 4B).

During the follow-up period, no significant difference in the AS length between right eyes (5144.5 µm [4151.5; 7821]) and left eyes (median 5000 µm, IQR 3434; 7605.9) was present (*p* = 0.28). However, the eyes with ODD showed significantly faster AS growth at the follow-up visit (median 8677, IQR 5383; 10925) compared to the eyes without ODD (median 4810, IQR 3950; 6375).

The median AS growth per year in the overall cohort was 34.2 µm per year [−25.5; 130.8]. While the eyes without any ODD showed a progression of only 4.3 µm/year, the eyes with ODD (at any visit, BL or FU) exhibited a growth rate of 338.8 µm/year.

### 3.4. Retinal Atrophy Association with Optic Disc Drusen

At baseline, the median size of the atrophy was 2.53 mm^2^ [IQR 0; 13.31] in the right eyes and 1.64 mm^2^ [IQR 0; 8.6] in the left eyes. No association between retinal atrophy and the presence of ODD was found (*p* = 0.27).

This exemplary 55-year-old female PXE patient exhibited a visual acuity of 20/20 in her right eye. The characteristics of PXE, including the presence of optic disc drusen (ODD), angioid streaks (ASs) and pattern dystrophy, are shown in the FAF image (A). The corresponding visual field (B, Zeiss Humphrey HFA 3/860 Field Analyzer, SITA Fast, 24-2, III, white, 31.5 asb) showed an enlarged blind spot and an inferior arcuate scotoma (VFI24-2: 81%, MD-24-2: −6.57 dB). The OCT B-scan (C) confirmed the beginning of atrophy and a retinal epithelium detachment. Differentiation of scotoma due to retinal atrophy or glaucomatous damage is challenging.

## 4. Discussion

To the best of our knowledge, this is the first study correlating the presence of ODD in PXE with the angioid streak length as a surrogate for BrM calcification. In our cohort, we found a prevalence of 30.7% for ODD in PXE patients, a notably higher rate compared to the general population and above the previously reported prevalences ranging from 6–20% [12,13]. The preferred nasal localization of ODD aligns with previous studies [7].

ODD are more prevalent in PXE patients compared to populations with other ocular conditions. The exact cause of this remains unknown. The hallmark pathological feature in PXE is calcification of BrM, starting around the optic nerve head and extending toward the periphery. Our study revealed a correlation between the AS length and the presence of ODD. Furthermore, eyes with ODD showed a significantly faster AS growth per year. Thus, the increased prevalence of ODD is hypothetically explained by the same mechanism leading to BrM calcification.

Histopathologically, the lamina cribosa might be affected by calcification (similar to Bruch’s membrane), causing a slowed axoplasmic flow [14]. Notably, eyes with ASs were reported to have a shallower anterior lamina cribrosa depth and thinner lamina cribrosa thickness using spectral-domain OCT-enhanced depth imaging [15]. This could possibly impact the retrograde axonal transport, resulting in a predisposition for the development of ODD and even higher vulnerability to glaucomatous damage. The lamina cribrosa constitutes the posterior part of the sclera, which is fenestrated to allow the optic nerve to exit the eye. Histologically, these fenestrated connective tissue sheets mainly consist of interwoven skeins of collagen fibers arranged around the canals. Furthermore, the scleral portion of the lamina cribrosa in particular contains dense elastic fibers [16]. A recent study correlating retinal histological findings in human PXE donor eyes with clinical retinal imaging indicated that calcification involves both elastic fibers and collagen layers of BrM, with gradual change toward the periphery [5]. A recent histological study with 8 eyes from a 4 abcc6^−/−^ rat model showed nodular and linear calcification at the level of Bruch’s membrane. Furthermore, the histological examination revealed foci of calcification in the uveal tract stroma, in the sclera, and in the conjunctiva [17]. From this, it could be postulated that calcification may also impact the lamina cribrosa and its aforementioned layers. Histopathological examinations, including investigation of the lamina cribrosa in PXE, would be interesting to validate these considerations.

Furthermore, ODD were only visible beyond an AS length of 5000 µm. These findings—although based on cross-sectional data—suggest a temporal sequence of ODD following AS development and progression to an intermediate stage. Future prospective analyses are necessary to monitor the development and progression of ODD and to identify the threshold of calcification at which ODD arise, although this is difficult due to the slow progression of changes.

ODD have been reported to be associated with both morphological degeneration (i.e., thinning of the ganglion cell layer and nerve fiber layer) and dysfunction (i.e., visual field defects), although to a variable extent. Sato et al. [18] observed that larger ODD are associated with significant abnormalities in the retinal nerve fiber layer in ODD eyes in general. Specifically for PXE, our group has identified ODD as a risk factor for inner retinal degeneration, suggesting that mechanical stress, along with ODD as an enhancing factor, leads to retrograde axonal degeneration and thinning of the ganglion cell layer (GCL) [19,20].

Visual field loss (arcuate field defects, enlarged blind spots, nasal steps, and constricted visual fields) is a common clinical finding in patients with ODD. Buried ODD usually show no or only small defects, while visible ODD and also with age visual field defects become more severe [9,21]. A long-term follow-up study showed that progression in visual fields in patients with ODD can begin as early as childhood [22]. During the lifetime, the rate of visual field loss progression in patients with ODD seems to become more severe with age [23]. OCT-based analysis showed that retinal nerve fiber layer thinning was associated with the ODD volume and also the nasal localization of ODD [24,25].

Interestingly, the ODD were independent of the area of outer retinal atrophy in our study, suggesting that the outer degenerative process might be governed by multifactorial pathways additional to the bare BrM calcification.

**Figure 5:**
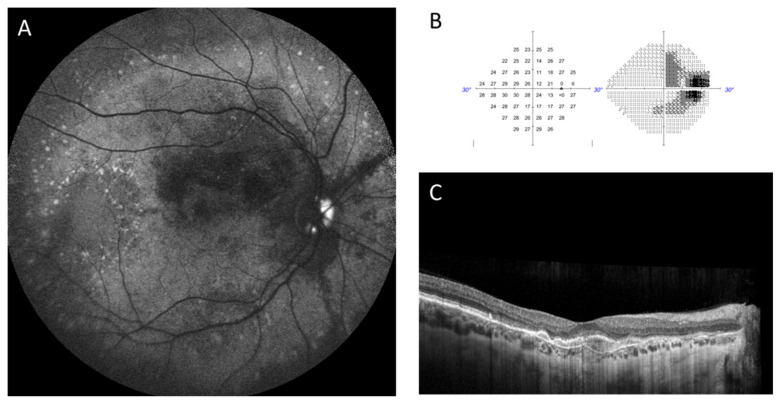
Fundus autofluorescence (FAF) imaging (**A**), visual field examination (**B**) and optical coherence tomography (OCT) B-scan (**C**) to illustrate the morphological and possible functional aspects of optic disc drusen (ODD).

The development and progression of ODD in our cohort seem to be slow, as only one patient developed ODD in our observational cohort. In PXE, macular neovascularization, exudation and, in late stages, subretinal fibrosis or outer retinal atrophy are very frequent. Therefore, attributing visual field defects to either outer retinal changes (i.e., retinal atrophy and fibrosis) or retinal nerve fiber damage due to ODD can be challenging in very progressed stages (see Figure 5). We recommend monitoring PXE patients with ODD regularly for visual field defects and including imaging of the retinal nerve fiber layer for detection of early degeneration. In case of elevated intraocular pressure (IOP), topical IOP lowering drugs may be considered to slow the retinal nerve fiber atrophy. This decision should consider various risk factors, including age, indicators of progression, and contraindications.

### Limitations

Our study has limitations. First, small or buried ODD may not be visible on FAF imaging, leading to a possible under-reporting in our analysis. For these, ultrasonography or enhanced depth-imaging OCT could be an additional diagnostic tool. Second, in case of peripapillary atrophy, the optic disc rim was difficult to assess for determination of the AS length. Also, in cases of advanced disease stage, atrophy overlays ASs, making them challenging or impossible to measure, which is also reflected in some negative measurements of the AS progression. In these cases, infrared-reflectance imaging or fundus photographs could be helpful to assess the AS length. Furthermore, functional testing such as visual field testing was only performed at selected visits. A prospective study would be needed to assess the possible visual field defects and their progression.

## 5. Conclusions

To summarize, in our study, ODD are evident in almost every third PXE patient (30.7%), which is markedly more frequent than in the general population (~2.4%). The association of a more extensive AS length (an indicator of the state and extent of ocular BrM calcification) and ODD suggests that ODD are associated with the degree of ectopic calcification, possibly affecting the lamina cribrosa. Further prospective studies are necessary to determine the impact of ODD on visual function in PXE and whether the IOP—a potential treatment target—modifies this relationship.

## Data Availability

Data available on request (K.P.) due to privacy/ethical restrictions.

## References

[1] Bergen A.A., Plomp A.S., Schuurman E.J., Terry S., Breuning M., Dauwerse H., Swart J., Kool M., Van Soest S., Baas F. (2000). Mutations in ABCC6 cause Pseudoxanthoma elasticum. Nat. Genet..

[2] Le Saux O., Urban Z., Tschuch C., Csiszar K., Bacchelli B., Quaglino D., Pasquali-Ronchetti I., Pope F.M., Richards A., Terry S. (2000). Mutations in a gene encoding an ABC transporter cause Pseudoxanthoma elasticum. Nat. Genet..

[3] Jansen R.S., Küçükosmanoğlu A., de Haas M., Sapthu S., Otero J.A., Hegman I.E.M., Bergen A.A.B., Gorgels T.G.M.F., Borst P., van de Wetering K. (2013). ABCC6 prevents ectopic mineralization seen in Pseudoxanthoma elasticum by inducing cellular nucleotide release. Proc. Natl. Acad. Sci. USA.

[4] Gliem M., Müller P.L., Birtel J., Hendig D., Holz F.G., Charbel Issa P. (2016). Frequency, Phenotypic Characteristics and Progression of Atrophy Associated With a Diseased Bruch’s Membrane in Pseudoxanthoma Elasticum. Investig. Ophthalmol. Vis. Sci..

[5] Risseeuw S., Pilgrim M.G., Bertazzo S., Brown C.N., Csincsik L., Fearn S., Thompson R.B., Bergen A.A., Brink J.B.T., Kortvely E. (2024). Bruch’s Membrane Calcification in Pseudoxanthoma Elasticum: Comparing Histopathology and Clinical Imaging. Ophthalmol. Sci..

[6] Charbel Issa P., Finger R.P., Götting C., Hendig D., Holz F.G., Scholl H.P.N. (2010). Centrifugal fundus abnormalities in Pseudoxanthoma elasticum. Ophthalmology.

[7] Pipelart V., Leroux B., Leruez S., Henni S., Navasiolava N., Martin L., Ebran J.-M. (2019). A study of optic nerve head drusen in 38 Pseudoxanthoma elasticum (PXE) patients (64 eyes). Location of optic nerve head drusen in PXE. J. Fr. Ophtalmol..

[8] Palmer E., Gale J., Crowston J.G., Wells A.P. (2018). Optic Nerve Head Drusen: An Update. Neuroophthalmology.

[9] Auw-Haedrich C., Staubach F., Witschel H. (2002). Optic disk drusen. Surv. Ophthalmol..

[10] Hess K., Gliem M., Issa P.C., Birtel J., Muller P.L., von der Emde L., Herrmann P., Holz F.G., Pfau M. (2020). Mesopic and Scotopic Light Sensitivity and Its Microstructural Correlates in Pseudoxanthoma Elasticum. JAMA Ophthalmol..

[11] Plomp A.S., Toonstra J., Bergen A.A.B., van Dijk M.R., de Jong P.T.V.M. (2010). Proposal for updating the Pseudoxanthoma elasticum classification system and a review of the clinical findings. Am. J. Med. Genet. A.

[12] Pierro L., Brancato R., Minicucci M., Pece A. (1994). Echographic diagnosis of Drusen of the optic nerve head in patients with angioid streaks. Ophthalmol. J. Int. D’ophtalmologie Int. J. Ophthalmol. Z. Augenheilkd..

[13] Finger R.P., Charbel Issa P., Ladewig M., Götting C., Holz F.G., Scholl H.P.N. (2009). Fundus autofluorescence in Pseudoxanthoma elasticum. Retina.

[14] Coleman K., Ross M.H., Mc Cabe M., Coleman R., Mooney D. (1991). Disk drusen and angioid streaks in Pseudoxanthoma elasticum. Am. J. Ophthalmol..

[15] Demir G., Altan C., Cakmak S., Topcu H., Yasa D., Demircan A., Alkin Z. (2019). Evaluation of lamina cribrosa in angioid streaks using spectral-domain optical coherence tomography enhanced depth imaging. J. Fr. Ophtalmol..

[16] Elkington A.R., Inman C.B., Steart P.V., Weller R.O. (1990). The structure of the lamina cribrosa of the human eye: An immunocytochemical and electron microscopical study. Eye.

[17] Sehgal A., Milman T., Li Q., Pulido J.S. (2024). Histological Findings in the Eyes of Abcc6 Knockout Rat Model of Pseudoxanthoma Elasticum. Transl. Vis. Sci. Technol..

[18] Sato T., Mrejen S., Spaide R.F. (2013). Multimodal imaging of optic disc drusen. Am. J. Ophthalmol..

[19] Hess K., Raming K., Charbel Issa P., Herrmann P., Holz F.G., Pfau M. (2023). Inner retinal degeneration associated with optic nerve head drusen in Pseudoxanthoma elasticum. Br. J. Ophthalmol..

[20] Lee K.M., Woo S.J., Hwang J.-M. (2013). Morphologic Characteristics of Optic Nerve Head Drusen on Spectral-Domain Optical Coherence Tomography. Am. J. Ophthalmol..

[21] Wilkins J.M., Pomeranz H.D. (2004). Visual manifestations of visible and buried optic disc drusen. J. Neuro-Ophthalmol. Off. J. N, Am. Neuro-Ophthalmol. Soc..

[22] Malmqvist L., Hamann S. (2017). Photographic Documentation of Optic Disc Drusen Over More Than 50 Years. JAMA Ophthalmol..

[23] Lee A.G., Zimmerman M.B. (2005). The rate of visual field loss in optic nerve head drusen. Am. J. Ophthalmol..

[24] Tsikata E., Verticchio A.C.V., Falkenstein I., Poon L.Y.-C., Brauner S., Khoueir Z., Miller J.B., Chen T.C. (2017). Volumetric Measurement of Optic Nerve Head Drusen Using Swept-Source Optical Coherence Tomography. J. Glaucoma.

[25] Skaat A., Muylaert S., Mogil R.S., Furlanetto R.L., Netto C.F., Banik R., Liebmann J.M., Ritch R., Park S.C. (2017). Relationship Between Optic Nerve Head Drusen Volume and Structural and Functional Optic Nerve Damage. J. Glaucoma.

